# Endoscopic surgery for papillary thyroid microcarcinoma: cases review and investigation of technique dissemination in China

**DOI:** 10.3389/fendo.2025.1448672

**Published:** 2025-04-15

**Authors:** Yinghui Liang, Shuangta Xu

**Affiliations:** Thyroid and Breast Department, The Second Affiliated Hospital of Fujian Medical University, Quanzhou, China

**Keywords:** thyroid microcarcinoma, trans-breast, endoscopic thyroid surgery, trans-axillary, trans-oral

## Abstract

**Introduction:**

Thyroid cancer has become one of the most common types of cancer, with microcarcinomas accounting for more than 50% of all newly diagnosed thyroid cancers. Endoscopic thyroid surgery, which relocates the incision to a less visible area of the body, offers significant postoperative cosmetic benefits and has been widely used in the treatment of thyroid microcarcinomas. This study conducts a retrospective case analysis and questionnaire survey to provide clinical reference by statistically analyzing the development of endoscopic techniques in China over the past five years.

**Materials and methods:**

This study included cases of thyroid microcarcinoma surgeries performed in our hospital from January 2018 to March 2020. Postoperative follow-up was conducted using the THYCA-QoL questionnaire. A network questionnaire survey was carried out through the Chinese Society of Oncoplastic Endocrine Surgeons targeting its members and other institutions performing endoscopic thyroid surgeries. The data was analyzed to obtain relevant results.

**Results:**

The complications were comparable between the endoscopic and open surgery groups. The incidence of neck discomfort was higher in the open surgery group compared to the endoscopic group (21.92% vs. 13.38%). Scar formation was also more noticeable in the open surgery group (23.74% vs. 17.2%). The local recurrence rates were similar between the two groups (1.27% vs. 1.37%). Over 85% of the surveyed institutions reported endoscopic thyroid surgery via trans-breast approach, while the trans-axillary approach showed the fastest growth over the past five years. Most institutions (approximately 80%) performed fewer than 300 endoscopic thyroid surgeries annually. Additionally, in most institutions (around 67%), endoscopic thyroid surgeries accounted for less than 20% of the total thyroid surgeries performed annually. The most frequently questioned issues regarding the trans-breast and trans-axillary approaches were the completeness of central lymph node dissection (with 66.78% and 40.46% of respondents, respectively, considering the lymph node dissection to be incomplete). Furthermore, about 20%-30% of the surveyed institutions believed that endoscopic surgery was more traumatic and associated with a higher incidence of post-thyroidectomy syndrome (PTS).

**Conclusion:**

For papillary thyroid microcarcinoma, endoscopic surgery demonstrates comparable efficacy to traditional open surgery, with no significant differences observed in 5-year recurrence and survival rates during follow-up. However, the safety and reliability of various endoscopic approaches for thyroid cancer surgery remain questionable, particularly regarding the thoroughness of central compartment lymph node dissection, as indicated by surveys on the implementation of endoscopic thyroid surgery over five consecutive years. More long-term follow-up data are required to validate these outcomes. Therefore, we recommend that preoperative lymph node positivity should be considered a contraindication, and patients with postoperative pathological confirmation of lymph node metastasis warrant closer clinical monitoring and intensive follow-up.

## Introduction

The incidence of thyroid cancer has been continuously increasing in recent years (1), particularly in China, where it has become one of the most common types of cancer, accounting for 38% of the global annual new cases (2). Among these, papillary thyroid microcarcinoma (PTMC), which measures 1 cm or less in diameter, represents more than 50% of all new thyroid cancer cases. According to 2015 ATA guidelines (3), surgery remains the preferred treatment method for thyroid cancer. Compared to traditional open thyroid surgery, endoscopic surgery offers advantages such as less visible incisions and better cosmetic results. Endoscopic thyroid surgery has rapidly developed and gained widespread adoption in China (4). Over the past decade, advances in technology have led to the extensive clinical application of endoscopic thyroid cancer surgery. The current practice of endoscopic thyroid surgery emphasizes that clinicians should perform the procedure only after accumulating years of experience in both open and endoscopic surgeries, requiring a certain number of cases to gradually master and refine the technique. Kuo et al.’s (5) study demonstrated that the learning curve for neuromonitoring-assisted transoral endoscopic thyroidectomy vestibular approach (TOETVA) was 35 cases when performed by surgeons with extensive experience in open surgery and minimally invasive video-assisted thyroidectomy. Liang et al.’s (6) research found that surgeons with prior experience in axillo-breast approach surgery achieved significantly shorter operative times after completing 23 TOETVA cases. In previous studies by the surgical team involved in this research, it was observed that the first 38 cases represented the technical exploration phase, with technical proficiency achieved after accumulating 65 cases. As an emerging surgical technology of the 21st century, endoscopic thyroid surgery has gradually evolved towards maturity and standardization, leading to the development of various technical approaches. Additionally, the safety, reliability, and long-term follow-up of endoscopic thyroid cancer surgery have received increasingly objective evaluations in the studies (7, 8). This study retrospectively analyzes cases of endoscopic thyroid cancer surgery performed at a mature technical stage and uses an online survey to collect data on the implementation and common issues of endoscopic technology in China over the past five years, aiming to provide more objective references for clinical practice.

## Materials and methods

General Information: This study included 432 cases of thyroid microcarcinoma surgery performed in our hospital from January 2018 to March 2020 (**Table 1**). Inclusion criteria: 1. Age between 18 and 65 years. 2. Tumor size ≤1 cm. 3. Pathological diagnosis of thyroid micro-papillary carcinoma. 4. Imaging examinations (ultrasound, CT) indicating no signs of cervical lateral lymph node metastasis. 5. No hoarseness before surgery, and laryngoscopy showed no vocal cord paralysis. 6. No history of surgery or radiotherapy in the anterior neck or chest before surgery. Exclusion criteria: 1. Postoperative pathology indicating non-thyroid papillary carcinoma. 2. The pathology suggests microscopic capsular invasion or extrathyroidal extension. 3. Patients with other malignant tumors. 4. Patients who underwent conversion to open surgery due to severe intraoperative complications. After excluding 56 cases lost to follow-up, 376 cases were finally included, with 157 cases in the endoscopic group and 219 cases in the open surgery group. The median follow-up time was 65.2 ± 12.1 months.

**Table 1:** Statistical analysis of case grouping.

	Trans-breast approach (A)	Trans-axillary approach (B)	Open surgery (C)	(C) vs. (A+B)	
				t/x²	P-value
*Patients*	116 (all females)	41 (male:11, female:30)	219 (male:68, female:151)		
*Ages (years)*	41.3 ± 6.6	44.8 ± 7.2	48.2 ± 6.7		
*BMI*	23.6 ± 3.8	22.3 ± 5.6	24.1 ± 4.2	1.803	0.072
*Tumor size (cm)*	0.65 ± 0.32	0.57 ± 0.26	0.63 ± 0.33	0.617	0.538
*Bilateral tumors*	11	0	32	5.222	0.017
*Combined with Hashimoto’s thyroiditis*	21	11	47	0.064	0.799
*Lymph node harvest*	4.36 ± 0.56	3.8 ± 0.43	5.6 ± 0.63	23.231	0.00<0.05
*Lymph node metastasis*	16 (13.79%)	6 (14.63%)	52 (23.74%)	0.596	0.437
*Operation time (min)*	83.58 ± 26.32	92.36 ± 23.15	76 ± 24.56	-4.062	0.00<0.05
*Blood loss (ml)*	13.65 ± 5.86	12.78 ± 7.12	14.36 ± 5.12	1.787	0.075
*Drain time (days)*	2.23 ± 1.35	2.56 ± 1.46	2.43 ± 1.26	0.729	0.466
*Postoperative suction* *drainage (ml)*	55.36 ± 12.21	51.23 ± 17.36	45.57 ± 15.32	-5.142	0.00<0.05
*Hospital stay (days)*	3.21 ± 1.25	3.34 ± 1.36	3.28 ± 1.31	0.221	0.825

### Surgical methods

#### Open surgery group

Patients were placed in a supine position with upper limbs abducted and administered combined intravenous and inhalation anesthesia. A transverse cervical incision approximately 5 cm in length was made. For unilateral cases, a lobectomy and isthmusectomy were performed on the affected side along with a central compartment lymph node dissection. In patients with bilateral thyroid cancer, total thyroidectomy was conducted combined with bilateral central compartment lymph node dissection.

#### Trans-breast approach thyroid cancer radical surgery

**Figure 1:**
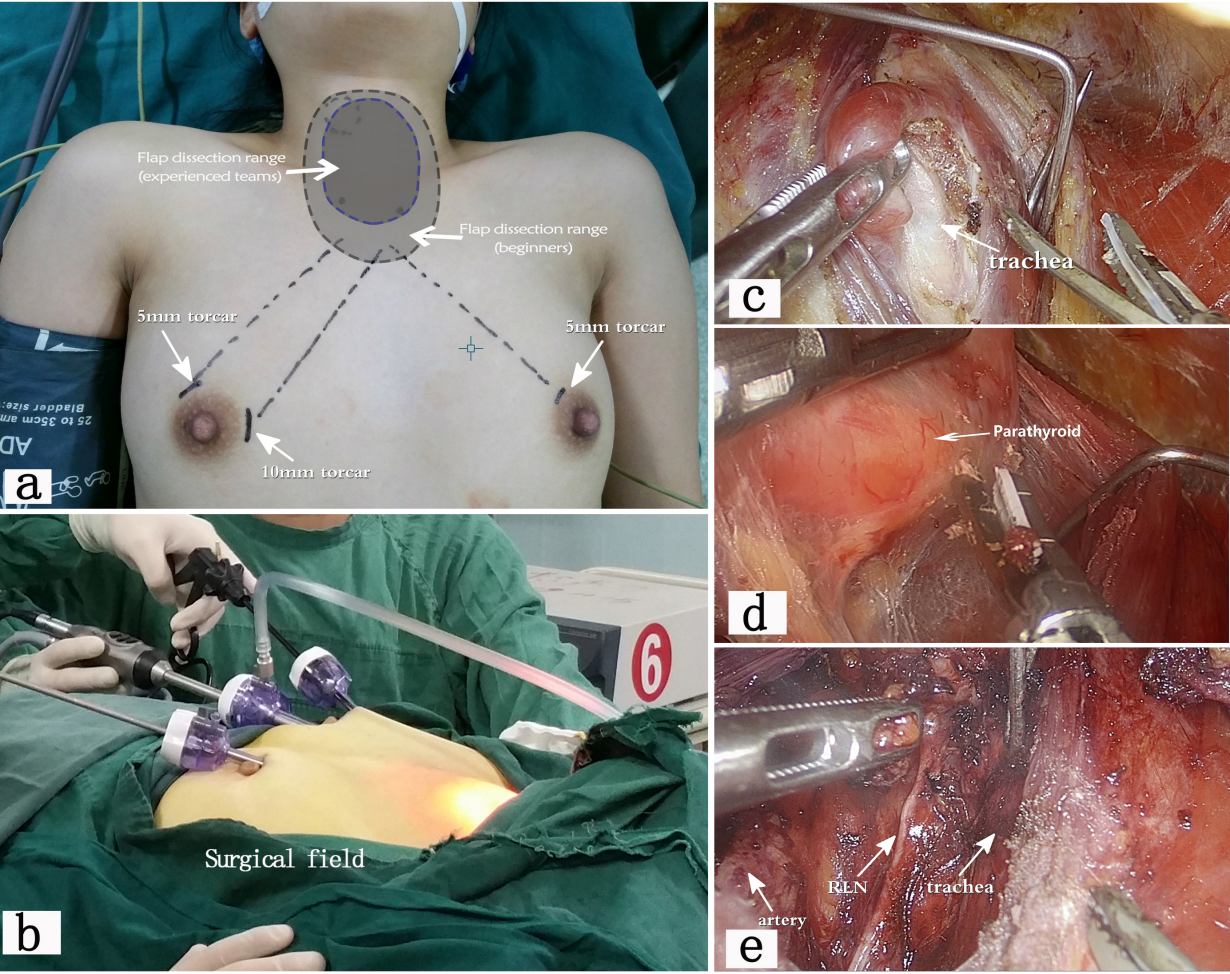
shows the procedure of endoscopic thyroid surgery via the trans-breast approach (12). **(a)** illustrates the surgical position and the location of the incision. Beginners often need to separate a larger flap area to establish a sufficiently operating space. Experienced teams require a smaller operating space, resulting in less postoperative trauma. **(b)** shows the operating caves and the external surgical field. **(c, d)** display the tissue structures under the endoscope, clearly showing the trachea, thyroid, parathyroid glands, and other tissues. **(e)** demonstrates the postoperative view after endoscopic unilateral thyroidectomy, with the recurrent laryngeal nerve (RLN)preserved intact.

As shown in **Figure 1**. The surgical position was the same as for open thyroid surgery, under general anesthesia. The procedure followed the “Guidelines for the Diagnosis and Treatment of Thyroid Nodules and Differentiated Thyroid Cancer”. Unilateral lobectomy with isthmectomy was performed, along with CLN dissection.

#### Trans-axillary approach thyroid cancer radical surgery

**Figure 2:**
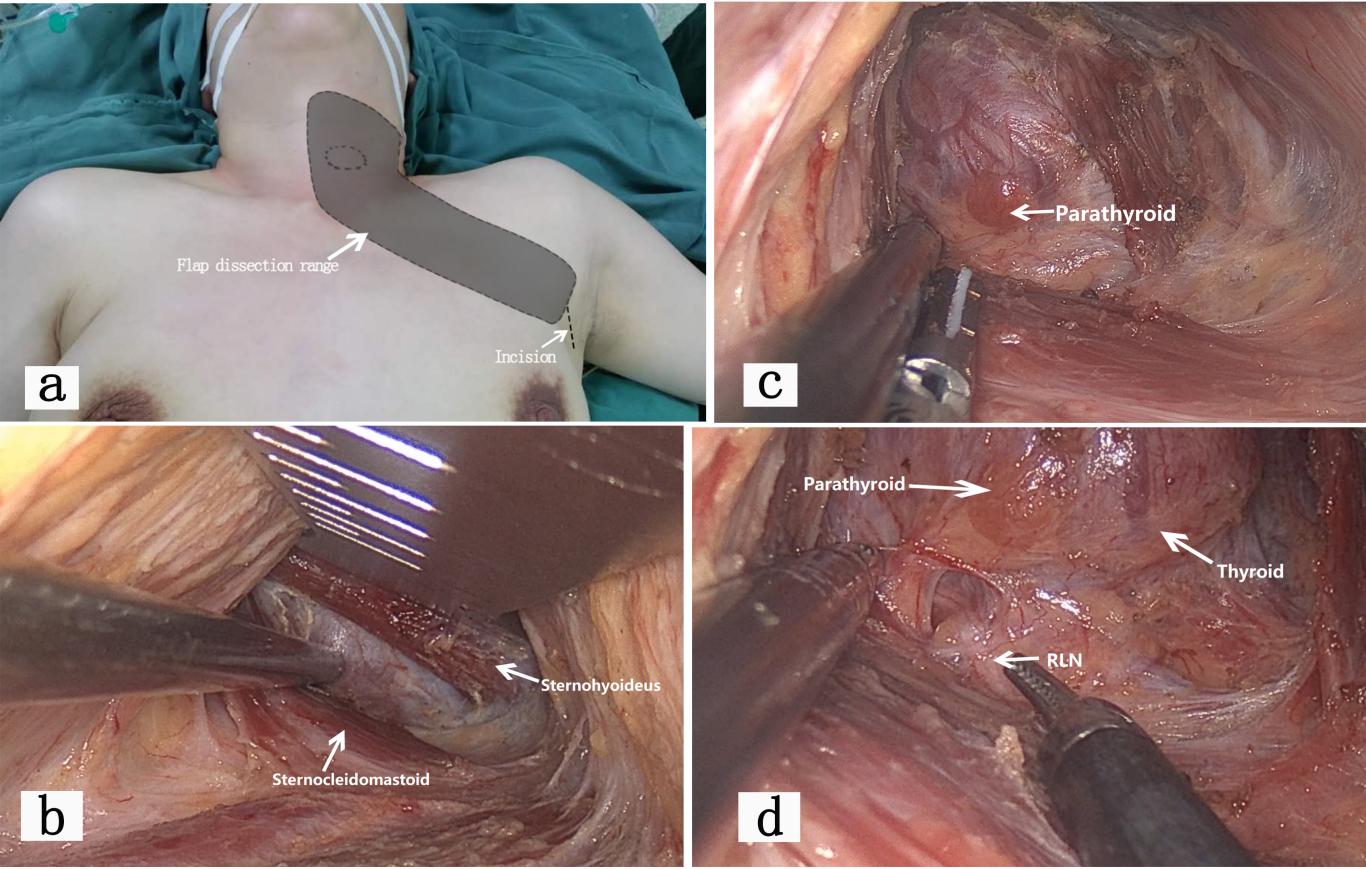
illustrates the entire process of trans-axillary approach endoscopic thyroid surgery. **(a)** shows the patient’s position, incision location, and flap dissection range for axillary approach endoscopic thyroidectomy. The overall trauma is larger than that of open surgery. This approach is suitable for patients with tumors located in one lobe of the thyroid. **(b)** shows the endoscopic view via the axillary approach. The dissection plane is located anterior to the pectoralis major fascia, creating a space between the sternal and clavicular heads of the sternocleidomastoid muscle until the sternohyoid muscle. The thyroid is exposed below the sternohyoid muscle. Compared to open surgery, the axillary approach does not damage the cervical cutaneous nerves, offers a deeper operating space, and results in fewer complications such as postoperative numbness and neck tightness. This finding was also supported by similar conclusions in related studies (13). However, care must be taken to protect the supraclavicular nerves. **(c, d)** show important anatomical structures visible through the endoscopic view of the axillary approach. With the magnification effect of the endoscope, the thyroid, parathyroid glands, and RLN are clearly visible. Due to the obstruction of the clavicle, the dissection of some CLNs may be limited.

As shown in **Figure 2**. The surgical position was supine with arms abducted, under general anesthesia. The procedure was the same as the chest-breast approach, performing unilateral lobectomy with isthmectomy and CLN dissection.

For bilateral thyroid cancer, total thyroidectomy with bilateral CLN dissection was performed. All patients were given liquid food 6 hours after surgery, semi-liquid food on the first postoperative day, and antibiotics were not routinely used. Patients were discharged when the single-day drainage volume was less than 20 ml.

#### Postoperative follow-up

All patients regularly took levothyroxine tablets after surgery. According to the ATA guidelines for thyroid cancer recurrence risk stratification, outpatient follow-up and TSH control were maintained at 0.1-0.5 mU/L. Follow-up examinations were conducted every 3-6 months. Six months after surgery, patients were interviewed using the Chinese version of the THYCA-QoL (9) questionnaire (**Table 2**).

**Table 2:** Complications and follow-up.

		Trans-breast approach (A)	Trans-axillary approach (B)	Open surgery (C)	(C)vs (A+B)P-value
*Complication*	Transient RLN injury	7 (6.03%)	3 (7.32%)	16 (7.31%)	0.723
	Permanent RLN injury	1 (0.86%)	1 (2.44%)	3 (1.37%)	0.705
	Skin flap ischemia	5 (4.31%)	1 (2.44%)	7 (3.2%)	0.742
	Subcutaneous hematoma	9 (7.76%)	2 (4.88%)	13 (5.94%)	0.674
	Numbness and pain	20 (17.24%)	6 (14.63%)	39 (17.81%)	0.751
	Bleeding requires reoperation	2 (1.72%)	0	3 (1.37%)	0.705
*Follow-up at 6 months after surgery.*	Unilateral vocal cord paralysis	1 (0.86%)	1 (2.44%)	2 (0.91%)	0.862
	Discomfort with neck movement	16 (13.79%)	5 (12.2%)	48 (21.92%)	0.030
	Scar formation	22 (18.97%)	5 (12.2%)	52 (23.74%)	0.114
	Choking sensation when swallowing	8 (6.9%)	2 (4.88%)	23 (10.5%)	0.158
	A sense of dysphonia	7 (6.03%)	2 (4.88%)	13 (5.94%)	0.934
	Numbness and tingling in the skin of the neck and chest	8 (6.9%)	3 (7.32%)	15 (6.85%)	0.953
*Loco-regional recurrence*		2 (1.72%)	0	3 (1.37%)	0.705
*Mortality*		0	0	0	

### Survey on endoscopic thyroid surgery in china

#### Data source

A questionnaire was designed based on common approaches, implementation status, and common issues of endoscopic thyroid surgery. An online survey was conducted among members of the Chinese Society of Oncoplastic Endocrine Surgeons (CSOPES) and other centers that had performed endoscopic thyroid surgery.

#### Collection and organization of survey data

**Figure 3:**
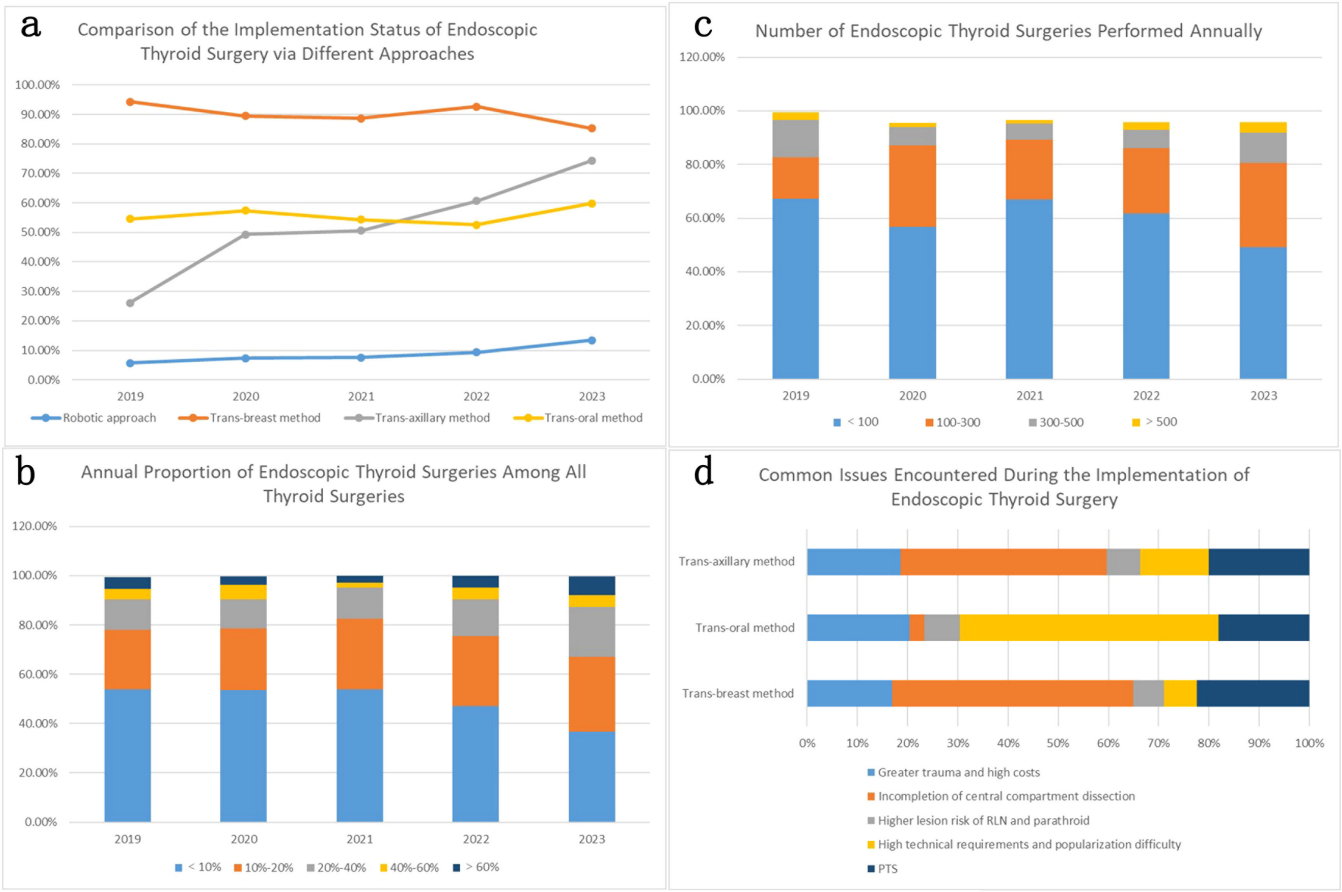
**(a)** shows the comparison of different approaches to endoscopic thyroid surgery over the past five years. The trans-breast approach is the most beginner-friendly and has become the most widely adopted (more than 85% of the surveyed institutions have implemented it) in China. The trans-oral approach, possibly due to its technical difficulty and lower acceptance, is only practiced in 50%-60% of the surveyed institutions. The trans-axillary approach has seen the fastest growth in the past five years, and by 2023, it was adopted by 74.34% of the surveyed institutions. With the advancement of technology and equipment, the number of institutions adopting the robotic approach is also gradually increasing. **(b, c)** respectively represent the number and proportion of endoscopic thyroid surgeries performed annually by the surveyed institutions. Approximately 50% of the institutions perform fewer than 100 endoscopic thyroid surgeries annually, and most institutions (about 80%) perform fewer than 300 cases per year. In most institutions (about 67%), endoscopic thyroid surgeries account for less than 20% of all thyroid surgeries performed annually. **(d)** summarizes the common issues encountered during the implementation of different approaches to endoscopic thyroid surgery. The most frequently questioned issue for both the trans-breast and trans-axillary approaches is the thoroughness of CLN dissection (66.78% and 40.46% of respondents respectively believe that lymph node dissection in the central neck area is not thorough with these approaches). The main issue with the trans-oral approach is its high technical difficulty and skill requirements. Additionally, about 20%-30% of the surveyed institutions believe that endoscopic surgery is more traumatic and has a higher incidence of post-thyroidectomy syndrome (PTS).

Data collection covered a 5-year period from 2019 to 2023 (**Table 3**). The survey included major provinces and cities nationwide, with over 85% of the participating centers being public tertiary hospitals. The results objectively reflect the current implementation status and common issues of endoscopic thyroid surgery (**Figure 3**).

**Table 3:** Survey on the implementation of endoscopic thyroid surgery in China.

		2019		2020	2021	2022	2023
Total questionnaire		295		312	342	447	304
Public tertiary hospital		265 (89.8%)		270 (86.54%)	303 (88.6%)	390 (87.25%)	262 (86.18%)
Trans-breast approach		278 (94.24%)		279 (89.42%)	303 (88.6%)	414 (92.62%)	259 (85.2%)
Trans-axillary approach		77 (26.1%)		154 (49.36%)	173 (50.58%)	271 (60.63%)	226 (74.34%)
Trans-oral approach		161 (54.58%)		179 (57.37%)	186 (54.39%)	235 (52.57%)	182 (59.87%)
Robotic approach		17 (5.76%)		23 (7.37%)	26 (7.6%)	42 (9.4%)	41 (13.49%)
Endoscopic approach rate	<10%	159 (53.9%)		167 (53.53%)	184 (53.8%)	211 (47.2%)	112 (36.84%)
	10%-20%	71 (24.07%)		78 (25%)	97 (28.63%)	127 (28.41%)	92 (30.26%)
	20%-40%	37 (12.54%)		37 (11.86%)	44 (12.87%)	66 (14.77%)	61 (20.07%)
	40%-60%	12 (4.07%)		18 (5.77%)	6 (1.75%)	22 (4.92%)	15 (4.93%)
	>60%	14 (4.75%)		11 (3.53%)	10 (2.92%)	21 (4.7%)	23 (7.57%)
Cases per year	<100	198 (67.12%)		177 (56.7%)	229 (66.96%)	276 (61.74%)	149 (49.01%)
	100-300	46 (15.59%)		95 (30.4%)	76 (22.22%)	109 (24.38%)	96 (31.58%)
	300-500	41 (13.9%)		21 (6.73%)	21 (6.14%)	30 (6.71%)	34 (11.18%)
	>500	8 (2.71%)		5 (1.6%)	4 (1.17%)	13 (2.91%)	12 (3.95%)
Disadvantages	Trans-breast method	Incompletion of CLN dissection (66.78%), Greater trauma and high costs (23.68%), High technical requirements and popularization difficulty (9.21%), Higher lesion risk of RLN and parathyroid (8.55%), Scar formation on the chest or areola (2.6%), PTS (31.25%)					
	Trans-oral method	Incompletion of CLN dissection (3.62%), Greater trauma and high costs (23.03%), High technical requirements and popularization difficulty (57.86%), Higher lesion risk of RLN and parathyroid (7.89%), PTS (20.39%)					
	Trans-axillary method	Incompletion of CLN dissection (40.46%), Greater trauma and high costs (18.42%), High technical requirements and popularization difficulty (13.49%), Higher lesion risk of RLN and parathyroid (6.58%), PTS (19.74%)					
	Robotic approach	High equipment requirements. Few centers have carried out this approach.					

#### Statistical analysis

The reporting of this study conforms to STROBE guidelines (10). Data were analyzed using SPSS 24. Categorical data are presented as cases, and continuous data with a normal distribution are presented as mean ± standard deviation. Categorical data are expressed as the number of cases (n) and were analyzed using the χ2 test or Fisher’s exact test. A P value of <0.05 was considered statistically significant.

#### Ethics and informed consent

This study was approved by the Ethics Committee of the Second Affiliated Hospital of Fujian Medical University (Approval No. 032). All patients signed informed consent forms before treatment, agreeing that their treatment history and follow-up results could be used for clinical statistical research. The study obtained the patients’ informed consent, and the collected case images were obtained with the patients’ informed consent, complying with the relevant requirements of the Declaration of Helsinki by the World Medical Association (11).

## Results

**Table 1** presents the baseline characteristics of enrolled patients, including age, gender, body mass index (BMI), tumor size, lymph node status, operative time, intraoperative blood loss, postoperative wound drainage, and hospital stay. The endoscopic group comprised 157 cases (11 males, 146 females) with a mean age of 42.6 ± 6.8 years, mean BMI of 23.3 ± 4.3 kg/m², mean tumor size of 0.61 ± 0.28 cm, and mean number of resected lymph nodes of 4.21 ± 0.48. Lymph node metastasis was identified in 22 cases (14.01%). The mean operative time was 86.47 ± 24.79 minutes, mean intraoperative blood loss 13.32 ± 6.13 mL, mean drainage duration 2.33 ± 1.38 days, mean postoperative drainage volume 53.76 ± 15.12 mL, and mean hospital stay 3.25 ± 1.27 days. Notably, the endoscopic group demonstrated a higher proportion of female patients and younger mean age compared to the open surgery group, particularly in the trans-breast approach subgroup which exclusively involved female patients. Among trans-breast approach cases, 11 (9.48%) were bilateral thyroid cancer patients versus 32 (14.61%) in the open surgery group. The open surgery group exhibited greater central neck lymph node dissection volume and higher lymph node detection rates. Of particular interest, the lymph node positivity rate was higher in the open surgery group (23.74% vs 14.01%), potentially associated with stricter inclusion criteria for endoscopic procedures, though this difference had no statistical significance (P=0.437). The endoscopic group showed longer mean operative time and greater postoperative drainage volume compared to the open surgery group (P<0.05), while maintaining comparable tumor size, intraoperative blood loss, drainage duration, and hospital stay.

**Table 2** shows the postoperative complications and follow-up results. It can be seen that the incidence of postoperative RLN injury, flap ischemia, subcutaneous hematoma formation, local numbness and pain, and postoperative bleeding requiring reoperation for hemostasis are comparable between the endoscopic group and the open surgery group. In the follow-up survey at 6 months post-surgery, the incidence of neck discomfort (such as neck tightness, restricted neck movement, and swallowing difficulty) was higher in the open surgery group than in the endoscopic group (21.92% vs 13.38%, P<0.05). Regarding scar formation, according to the POSAS (14) assessment scale, the patient scale involves six aspects such as color, pliability, thickness, relief, Itching, and pain sensation, while the observer scale involves five aspects such as vascularization, pigmentation, pliability, thickness and relief. The open surgery group also had a higher incidence (23.74% vs 17.2%, P>0.05), with the trans-axillary approach having the lowest rate of scar formation (12.2%, P<0.05). During the follow-up, only 5 cases experienced local recurrence, with 2 cases in the endoscopic group and 3 cases in the open surgery group, indicating comparable local recurrence rates between the two groups (1.27% vs. 1.37%). No deaths were reported among the follow-up cases.

The results shown in **Table 3** are from a survey on the implementation of endoscopic thyroid surgery in China over a five-year period from 2019 to 2023. The number of valid questionnaires received were 295 in 2019, 312 in 2020, 342 in 2021, 447 in 2022, and 304 in 2023. The survey results include the main participating centers, the implementation status of several common approaches to endoscopic thyroid surgery, the proportion of endoscopic thyroid surgeries, the number of endoscopic thyroid surgeries performed annually, and the common issues encountered with several common approaches. The detailed data comparison is shown in **Figure 3**.

## Discussion

Development of Endoscopic Thyroid Surgical. Since Gagner (15) performed the first endoscopic parathyroidectomy via a cervical approach using insufflation in 1996, endoscopic thyroid surgery has undergone nearly 30 years of development and evolution. The greatest advantage of endoscopic techniques is the relocation of the incision to a more concealed area of the body, providing excellent cosmetic outcomes. Consequently, many scholars have experimented with different approaches to achieve this goal. Reported approaches (16) include trans-breast, trans-axillary, trans-retroauricular, trans-oral, trans-subclavian, and the Miccoli procedure. With advancements in technology and the accumulation of surgical experience, the trans-breast, trans-axillary, trans-oral, and robot-assisted approaches have gradually become mainstream and widely adopted due to their respective advantages (17). Our investigation into the implementation of endoscopic thyroid surgery over the past five years revealed that the trans-breast approach is currently the most widespread due to its relatively low technical difficulty, which facilitates surgeons’ proficiency and promotion. The trans-axillary approach, with its incision in the axilla, offers better concealment (18). Using suspension retractor devices to maintain space and suction devices to remove smoke promptly facilitates cavity creation and surgical operations, leading to its rapid promotion and adoption. The greatest advantage of the trans-oral approach is the absence of visible postoperative scars (as the incision is within the oral cavity), but its high surgical difficulty and the disadvantage of a non-sterile oral incision have limited its promotion. Robot-assisted thyroid surgery (19) offers a visual field similar to open surgery and is not restricted by the angle of surgical instruments, allowing for a direct visual resection range with excellent application prospects. However, the high equipment requirements have limited its rapid promotion. Currently, robot-assisted thyroid surgery is performed in only a few centers, and the number of accumulated clinical cases is slowly increasing.

Common Issues in Endoscopic Thyroid Surgery. Endoscopic surgical approaches require creating a surgical operating space through relatively long paths and freeing a sufficient area of skin flap to complete the surgery, which results in greater trauma compared to open surgery. This is consistent with our survey results, where approximately 20%-30% of scholars believe that the incidence of post-thyroidectomy syndrome in the flap area is higher after endoscopic thyroid surgery. Postoperative complications (20) can include numbness, swelling in the surgical area, and, in some cases, skin bruising and hematoma. Severe cases may require a second surgery due to skin flap bleeding. However, experienced teams can reduce skin complications in the surgical area by improving surgical details, such as minimizing the flap dissection range, precisely controlling the surgical layer, avoiding injury to the muscular membrane structures, and rationally using energy instruments (ultrasonic scalpel, electrocoagulation hook). Since we began performing endoscopic thyroid surgery in 2012, we have observed that, with increasing proficiency, the postoperative complications of endoscopic thyroid surgery are comparable to those of traditional open surgery. Follow-ups have shown that patients experience less foreign body sensation and scar retraction in the neck area post-endoscopic surgery compared to open surgery, likely due to the higher incidence of scars and adhesions in the surgical area in open surgeries. Compared to the trans-breast approach, the trans-axillary approach, when using specific auxiliary instruments, does not require the use of CO2 to create the surgical space (21). This results in no local subcutaneous emphysema and lighter discomfort in the surgical area. However, the axillary approach, due to the continuous traction on the sternocleidomastoid muscle by special instruments and the burns caused by energy devices during surgery, is prone to causing sternocleidomastoid muscle injury (22). The symptoms manifest as swelling, stiffness, and discomfort in the sternocleidomastoid muscle 2 weeks to 2 months post-surgery. These symptoms significantly alleviate or disappear within six months post-surgery. Our investigation revealed that some scholars believe endoscopic thyroid surgery is more likely to cause RLN and parathyroid injuries. While the endoscopic system offers a magnified view that provides an advantage in anatomical exposure compared to open surgery, beginners unfamiliar with the endoscopic approach, aerosol generation during ultrasonic scalpel use, and inadvertent injury by energy instruments can increase the risk of damage to the RLN and parathyroid glands. These risks decrease significantly with improved proficiency and teamwork coordination. Beginners can adopt intraoperative nerve monitoring and negative imaging of the parathyroid glands to reduce the risk of intraoperative nerve and parathyroid gland injuries.

Safety of Endoscopic Thyroid Surgery. Endoscopic thyroid surgery is considered a cosmetic procedure rather than a minimally invasive one. Thus, cosmetic needs are the primary motivation for choosing endoscopic thyroid surgery. However, the procedure involves a limited operating space and requires a highly skilled surgical team, necessitating stricter selection of surgical indications. Our survey result shows that, in most centers, endoscopic thyroid surgeries account for less than 20% of cases, with fewer than 100 cases performed annually. The reliability of CLN dissection via endoscopic surgery remains questioned by many scholars. Our investigation revealed that 66.78% of scholars using the trans-breast approach and 40.46% of those using the trans-axillary approach believe that CLN dissection is incomplete. Some studies (23, 24) report that the recurrence and survival rates of endoscopic thyroid cancer surgery are comparable to those of open surgery, with clinical reports available on the 10-year survival data post-endoscopic thyroid cancer surgery (25). In our practical experience, we found that some CLNs are located behind the sternum, creating visual blind spots in the trans-breast approach. Local traction during surgery can remove lymphatic tissue located behind the sternum, but whether the dissection is thorough cannot be confirmed visually. Similarly, the trans-axillary approach has visual blind spots obstructed by the sternum and clavicle. This is one of the main reasons many scholars believe this approach results in incomplete lymph node dissection. Therefore, we continue to insist on a detailed preoperative evaluation of neck lymph nodes when selecting indications. Cases with tumors confined to the thyroid and no imaging evidence of lymph node metastasis are chosen as indications for endoscopic thyroid surgery. Follow-ups of postoperative cases have shown no difference in recurrence and survival rates between the endoscopic and open surgery groups. Further data and long-term follow-up are still needed to support these findings. According to our survey on the application of endoscopic thyroid surgery, traditional endoscopic approaches face limitations in surgical maneuverability due to restricted operational space and visual blind spots, raising clinical concerns about the thoroughness of central compartment lymph node dissection under endoscopy. While the trans-oral approach addresses the retrosternal visual blind spots inherent in trans-breast and trans-axillary approaches, its technical complexity and increased infection risk have hindered widespread adoption (26). Robot-assisted thyroid surgery, leveraging the flexibility of robotic arms to overcome the practical limitations of conventional endoscopy, has gained increasing clinical utilization. Existing studies (27, 28) have evaluated the safety, reliability, and complications of robotic thyroid surgery, demonstrating positive outcomes that support broader clinical implementation and further in-depth research.

Considerations on the Application of Endoscopic Thyroid Cancer Surgery. Papillary thyroid carcinoma with lymph node metastasis is the main cause of recurrence (29). Many studies (30–32) have reported that selecting suitable cases for radical thyroidectomy under endoscopy, after careful preoperative evaluation, yields similar efficacy to open surgery. Our retrospective analysis shows that a portion of cases that were preoperatively assessed as lymph node-negative had postoperative pathological results indicating lymph node positivity (13.79% in the endoscopic group). Fortunately, these patients did not exhibit a higher recurrence rate compared to the open surgery group during long-term follow-up. According to the literature, Pacini (33) reviewed six studies on thyroid microcarcinoma, confirming that over 20% of thyroid microcarcinomas are multifocal, with 11% showing extrathyroidal invasion and 28% presenting with lymph node metastasis at the time of diagnosis. Roti et al. (34) included data from 17 articles and over 9,300 patients with thyroid microcarcinoma, showing that 15.0% had lymph node metastasis at diagnosis. Compared to the open surgery group, endoscopic surgery has stricter inclusion criteria, which may contribute to the lower lymph node positivity rate in the endoscopic group. Notably, the 2015 ATA guidelines (3) have further relaxed the indications for lobectomy. For low-risk differentiated thyroid cancer (meeting all of the following conditions: no significant extrathyroidal invasion, no neck lymph node involvement or distant metastasis, no family history of thyroid cancer, no history of head and neck radiation, and age ≤45 years), a tumor diameter of <4 cm is sufficient for lobectomy. For thyroid microcarcinoma, careful preoperative evaluation may indicate that most patients only need lobectomy. However, whether unilateral lobectomy is feasible for microcarcinoma patients with lymph node positivity requires more clinical data and longer follow-up to further prove its safety and reliability. Therefore, we believe that both beginners and experienced teams should adhere to the principle of treatment first. With the premise of strong cosmetic demands from patients, it is essential to carefully evaluate the condition, choose the appropriate surgical approach, and closely monitor the disease to achieve a balance between disease eradication, functional preservation, and cosmetic outcomes.

Limitations of This Study. 1. This study is a retrospective clinical case analysis. There is a selection bias between the endoscopic group and the open group among the enrolled cases, and the results obtained have clinical reference value under certain conditions. 2. The units participating in the questionnaire survey are mainly tertiary public hospitals, and some teams are still in the initial stage of performing endoscopic procedures. As proficiency increases and teams mature, some opinions may change. However, the overall survey results can objectively reflect the current prevalence and issues of endoscopic techniques. 3. According to reports, the average 5-year survival rate of papillary thyroid carcinoma in Japan is as high as 96.4%, with 98.1% for stage I and 98.6% for stage II (35). In the United States, the average 5-year survival rate for papillary thyroid carcinoma is reported to be over 99.5%, with over 99.5% for early stages and 99% for intermediate stages (36). During follow-up, we found that only 5 patients (2 in the endoscopic group and 3 in the open surgery group) experienced local recurrence, and none of the patients died. However, the 5-year follow-up data cannot truly reflect the differences in postoperative survival rates between the endoscopic and open surgery groups, requiring longer follow-up data. 4.The results of this survey reflect the application status and development trends of different approaches to endoscopic surgery in recent years, and also summarize the controversial issues that scholars are currently concerned about, which require further research.

## Conclusion

For papillary thyroid microcarcinoma, endoscopic surgery demonstrates comparable efficacy to traditional open surgery, with no significant differences observed in 5-year recurrence and survival rates during follow-up. However, the safety and reliability of various endoscopic approaches for thyroid cancer surgery remain questionable, particularly regarding the thoroughness of central compartment lymph node dissection, as indicated by surveys on the implementation of endoscopic thyroid surgery over five consecutive years. More long-term follow-up data are required to validate these outcomes. Therefore, we recommend that preoperative lymph node positivity should be considered a contraindication, and patients with postoperative pathological confirmation of lymph node metastasis warrant closer clinical monitoring and intensive follow-up.

## Data Availability

The original contributions presented in the study are included in the article/supplementary material. Further inquiries can be directed to the corresponding authors.
